# Cutaneous Hypersensitivity Dermatoses in the Feline Patient: A Review of Allergic Skin Disease in Cats

**DOI:** 10.3390/vetsci4020025

**Published:** 2017-05-09

**Authors:** Alison Diesel

**Affiliations:** Department of Small Animal Clinical Sciences, College of Veterinary Medicine and Biomedical Sciences, Texas A&M University, 4474 TAMU, College Station, TX 77843, USA; adiesel@cvm.tamu.edu; Tel.: +1-979-845-2351

**Keywords:** feline allergy, cat allergy, hypersensitivity, allergic skin disease, non-flea non-food hypersensitivity dermatitis, feline dermatitis, atopy, atopic dermatitis

## Abstract

Feline allergic skin disease presents a unique set of challenges to the veterinary practitioner. Although there is some similarity to what is seen in the allergic canine patient, cutaneous hypersensitivity dermatoses in cats can manifest with strikingly different clinical signs, treatment options and outcomes, and secondary complications/disease entities. Additionally, less is known about the pathogenesis of feline allergic skin diseases, particularly “feline atopic syndrome” when compared to dogs or people. This article aims to review what is currently known in regards to allergic skin disease in the feline patient, with focus on non-flea, non-food hypersensitivity dermatitis.

## 1. Introduction

Although it is often noted throughout the veterinary profession that cats are not small dogs, this fact becomes particularly apparent with regards to the manifestation of feline dermatological abnormalities. Cats present a unique clinical entity in terms of their manifestation of inflammatory skin disease, particularly the cutaneous hypersensitivity dermatoses. Frequently, the allergic feline patient will present with at least one of the four common cutaneous reaction patterns indicative of pruritus and inflammation: head/neck/pinnal pruritus with excoriations, self-induced alopecia, miliary dermatitis, and/or eosinophilic lesions (including eosinophilic plaques, eosinophilic granulomas, and indolent ulcers) [1]. The reaction patterns themselves are just that: patterns. They do not represent a definitive diagnosis. Although feline cutaneous reaction patterns are often indicative of underlying allergic skin disease, other differentials need to be considered prior to arriving at a diagnosis of allergy. This should include parasitic and infectious etiologies commonly; based on history, other clinical signs present, and core dermatologic diagnostics, other differentials including genetic and neoplastic conditions may also necessitate consideration. However, once a diagnosis of allergy is made, the specific reaction pattern noted may help the clinician determine which manifestation(s) of allergy should garner preferential consideration.

As with dogs, hypersensitivity skin diseases in cats fall into three main categories; however, the nomenclature is a bit different. Flea (and other insect bite) bite hypersensitivity and food induced hypersensitivity dermatitis (cutaneous adverse food reaction) are similar between dogs and cats, but atopic dermatitis is where the two species separate. Due to the lack of conclusively demonstrated influence of IgE (immunoglobulin E) on disease pathogenesis [2], the term “non-flea, non-food hypersensitivity dermatitis” (NFNFHD) is preferred when discussing “feline atopic syndrome” [1]. In general, when allergic skin disease is compared across the two species, much less is known/documented in cats as opposed to dogs. This article aims to review what is currently known in regards to allergic skin diseases in the feline patient with particular focus on non-flea, non-food hypersensitivity dermatitis. A brief discussion of flea bite hypersensitivity (as well as other insect bite allergies) and food induced hypersensitivity dermatitis is also presented within. 

## 2. Flea Bite Hypersensitivity and Other Insect Bite Allergies

### 2.1. Flea Bite Hypersensitivity (Flea Allergy Dermatitis)

Flea allergy dermatitis (FAD) has historically been considered the most frequently diagnosed hypersensitivity condition in the cat [3]. Classically, the disease presents more commonly in flea endemic areas and may be seen as a sole entity or in conjunction with other allergic skin disease, most commonly, NFNFHD. Evidence of flea exposure including “flea dirt” or the presence of live fleas may or may not be apparent [4]. Although dorsal lumbosacral pruritus with miliary dermatitis is noted most commonly with flea bite hypersensitivity [1] other reaction patterns may also be observed, leading to difficulty distinguishing between strictly FAD and other hypersensitivity diseases in the feline patient. This may be further complicated by the fastidious grooming behavior in cats which can make identification of fleas nearly impossible, particularly when only a small population is present. Other dermatologic lesions which may be noted with FAD include eosinophilic plaques or ulcers, bilaterally symmetrical self-induced alopecia, ventral abdominal alopecia, or generalized pruritus [1]. One study identified a frequent occurrence of indolent lip ulceration in cats with experimentally induced FAD; the same has also been noted for the naturally occurring disease [5]. The fact that several of these cutaneous reaction patterns may be observed highlights the importance of ruling out other potential causes of pruritus such as FAD prior to arriving at a clinical diagnosis of NFNFHD in the cat.

As in the dog, flea bite hypersensitivity reflects a type I (immediate-type) and/or type IV (delayed-type) hypersensitivity reaction in the cat [6]. Intradermal tests and allergen-specific IgE serology tests are frequently positive in cats with FAD [7,8]; however, it should be noted that approximately 36% of cats without dermatologic abnormalities are positive to flea extract on allergy testing according to one study [9]. This confirms the fact that “allergy testing” is not a yes or no test for whether an animal is allergic or not, but rather supports clinical findings. While flea saliva is made up of a wide array of components, it appears that certain proteins are more allergenic than others. A vaccine protocol was cloned, developed and administered to laboratory cats with experimentally induced FAD using a salivary protein termed flea salivary antigen 1 (FSA1) and a DNA vaccine. Administration of the vaccine lead to improved clinical signs in these allergic cats [10]; this finding may help pave the way for more targeted immunotherapeutic interventions in clinical patients in the future. The mainstay of management for cats with flea bite hypersensitivity, however, relies on consistent and aggressive adulticidal flea prevention, along with additional anti-pruritic medications for times of disease flare. Various products are available for the feline patient including continuous release collars, long-lasting topical isoxazoline medications, and other topical and oral anti-parasitics. Especially with the changing climate trends, year-round flea prevention is recommended by many veterinary dermatologists to prevent confounding diagnoses. To help further minimize exposure for the cat with FAD, all in-contact animals (e.g., other household pets) should also receive consistent adulticidal flea prevention. Additionally, where flea infestation is a potential concern, environmental treatment for both juvenile and adult stages of the parasite are indicated. This may best be accomplished by a professional exterminator.

### 2.2. Mosquito Bite Hypersensitivity

A somewhat closely related hypersensitivity condition in the feline patient is mosquito bite hypersensitivity. Also a type I (immediate-type) hypersensitivity reaction, mosquito bite hypersensitivity shares several features with FAD. The most common lesion reported is miliary dermatitis; eosinophils are rather easily demonstrated on lesions of affected cats. Head, neck and pinnal pruritus are commonly reported as lesions often concentrate along the convex surface of the pinnae as well as along the bridge of the nose. Paw pads may occasionally be implicated in disease presentation as well [11]. While less commonly seen, other eosinophilic lesions such as granulomas and plaques have been demonstrated in affected cats [12]. The disease is more commonly reported in cats that venture outside in geographic regions where mosquitos are endemic.

The mainstay of therapy involves avoidance as much as possible. Ideally, affected cats should be kept indoor where mosquito exposure is decreased. It is important to note that many insect repellents designed for humans are not safe to use on cats (e.g., those products containing DEET). Permethrin-based products should also be avoided due to toxicity seen in the feline species [11]. The newer synthetic pyrethroid, flumethrin, has proven safety in cats and may be beneficial for managing mosquito bite hypersensitive patients. While not yet evaluated, isoxazolines may additionally provide repellency due to their wide spectrum of insecticidal activity. As with FAD, administration of anti-pruritic therapy may be indicated periodically for disease flare. Similar to the management for FAD, environmental control for mosquitos may be helpful. If possible, standing water should be eliminated to decrease mosquito breeding grounds. Professional exterminators may be helpful to address heavily infested regions.

## 3. Food Induced Hypersensitivity Dermatitis (Cutaneous Adverse Food Reaction; Food Allergy)

In a cat with non-seasonal pruritus, and where parasitic and infectious causes of pruritus have been ruled out, food induced hypersensitivity dermatitis should be a considered differential diagnosis. Although information sources disagree on the prevalence of cutaneous reactions to food in the feline patient, with incidence ranging from 1% to 6% of all feline dermatological abnormalities [3], most sources agree that the condition is uncommon [13]. This may represent a true finding or it may be that it is under-investigated and hence underdiagnosed. Anecdotally, however, cutaneous adverse food reactions (CAFR) may be more common in the cat as compared to the dog. As with dogs [14], the pathogenesis of feline cutaneous adverse food reactions is not fully understood. Immunologic type I hypersensitivity reactions have been reported as well as increases in allergen-specific IgE in feline serum following oral allergen exposure in cats with concurrent *Toxoplasma gondii,* lending concern for the role of endoparasitism in the development of food induced hypersensitivity dermatitis [15]. The involvement of food intolerance (abnormal physiologic response to food not due to an immunologic reaction) [14] may also contribute to the development of cutaneous adverse reactions to food in the feline patient. In some cases, only an association between dietary influences and pruritus can be established.

Non-seasonal pruritus is the hallmark of feline food hypersensitivity. The condition presents similarly to NFNFHD in the cat in that one or more cutaneous reaction patterns may be noted. Although other body regions may be affected and the pruritus may be considered generalized in some cats, several reports identify pruritus as often disproportionally severe and more commonly involving the head, neck, and pinnae in cats with CAFR [1,16]. Concurrent gastrointestinal abnormalities are variably noted which may include vomiting, diarrhea, or soft stool. One study has reported concurrent gastrointestinal abnormalities in one-third of cats presenting for cutaneous adverse reactions to food [17]. Another found the combination of both gastrointestinal abnormalities and dermatological disease to be highly suggestive of food sensitivity in the feline patient [18]. As with dogs and people, some cats with food sensitivity may also have other concurrent hypersensitivities including NFNFHD or FAD [19], and vice versa, cats with NFNFHD may also have concurrent food sensitivity. The exact percentage where this occurs is not known.

Prior to investigating CAFR in the feline patient, parasitic and infectious causes of pruritus should be ruled out. Additionally, adulticidal flea prevention should be instituted to address sole or concurrent FAD. With regards to testing for food sensitivity, the only accurate way to diagnose the condition is with a strict elimination diet trial followed by a provocative food trial to confirm the food sensitivity. Without dietary challenge following improvement with a novel protein or hydrolyzed diet, food sensitivity can only be presumed. Serum allergy testing for IgE antibodies and intradermal testing with food allergens has been investigated in both dogs and people; both tests have been shown to be inaccurate and insensitive in the dog [20,21]. Although serologic and intradermal testing for food sensitivity has not been evaluated in the cat, the use of these tests are not recommended based on the canine model.

As with dogs, the length recommended for the hypoallergenic diet trial and dietary choice varies among veterinary dermatologists. Evidence based review of several sources found that more than 80% of cats with CAFR showed improvement with dietary manipulation within 6 weeks, however extending the diet trial to 8 weeks lead to complete remission in approximately 90% of cats and dogs with food sensitivity [22]. Whether a home-cooked or commercial hydrolyzed or novel protein diet is used during the elimination trial is dependent on practitioner preference, owner feasibility, and patient compliance. However, the choice relies on accurate dietary history and avoiding food items to which the cat has previously been exposed. Additionally, common food allergens including beef, chicken, and fish [23] should be avoided. A recent study evaluated the feeding of poultry-based hydrolyzed diets to dogs with known chicken sensitivity; 40% of the chicken-sensitive dogs manifested with pruritic flares when fed the hydrolyzed chicken liver diet [24]. Although not specifically tested in cats, this finding should be considered when choosing between various commercially available hydrolyzed diets; ideally hydrolyzed versions of proteins to which the patient has already been exposed should also be avoided during the elimination trial. Whatever diet is recommended, it should be fed strictly and then followed with a challenge of the original diet. Food sensitivity is confirmed if pruritus returns after administration of the original diet. As noted previously, in cats where both CAFR and NFNFHD are present, pruritus may only be decreased as opposed to eliminated with a hypoallergenic diet. A relative level of improvement should be evaluated by both the owner and veterinarian before and after the elimination diet trial.

## 4. Non-Flea, Non-Food Hypersensitivity Dermatitis (Feline Atopic Syndrome)

The term “feline atopy” was first introduced into the veterinary literature in 1982 [25]. The designation of “feline atopic dermatitis” was used to describe a clinical syndrome in a group of feline patients with recurrent pruritus, positive reactions to several common environmental allergens on intradermal testing, and where other causes of pruritus (external parasites, infections) had been ruled out. Over the years, however, primarily due to the lack of conclusive IgE involvement in the disease process, the veterinary community has rather adopted the terminology of “non-flea, non-food hypersensitivity dermatitis” when referring to what was historically called feline atopic dermatitis (AD) or feline atopic syndrome [1]. As with canine AD, pruritus is a hallmark of feline NFNFHD; however, the pattern of lesion distribution is more variable in the cat.

With regards to the pathogenesis surrounding NFNFHD in cats, the disease does share some similarities to both human and canine AD. Although less well described in cats, genetic influence of disease development does seem possible as is true for dogs and people [26]. Exposure to environmental allergens appears to exacerbate the disease in cats [27], but the role of allergen-specific IgE involvement in NFNFHD is still uncertain. Although the exact immunologic mechanism of disease development remains a bit unclear in the cat, histopathological studies have found a very similar pattern of inflammatory cells in the skin of cats with NFNFHD as compared to chronic AD lesions in dogs and people [28]. While T cell involvement also appears to be part of the immunopathogenesis of feline NFNFHD as shown by increased proportion of CD4+ T cells in the skin of allergic cats [29], cytokine profiles have not yet been evaluated. Whether or not interleukin-31 is involved in feline NFNFHD remains yet to be determined. The role of skin barrier function has become an increasingly important area of investigation with regards to human and canine AD. This has not been well evaluated in the cat.

Clinical signs of NFNFHD in cats revolve around the presence of pruritus. Lesion/pruritus distribution may include any one or more of the four cutaneous reaction patterns. As NFNFHD is a diagnosis of exclusion, as is true for AD in dogs and other species, ruling out parasites, infections, and other allergic diseases (FAD, CAFR) is imperative. In dogs and people, concurrent bacterial pyoderma secondary to underlying AD is considered to be a very common finding. The role of staphylococcal infections with regards to epidermal barrier function and the pathogenesis of AD in people has been well evaluated for several years; findings from various studies have changed the overall understanding of the disease and focus of therapeutic recommendations [30]. As dogs with AD have been shown to have a higher occurrence of superficial bacterial skin infections and higher skin surface counts of staphylococcal bacteria than healthy dogs, it is suspected that bacterial involvement in the pathogenesis of AD may be similar for both dogs and people [31]. Historically, concurrent bacterial pyoderma has been considered to be an uncommon to rare finding in cats with NFNFHD [31,32]. This may be due to decreased bacterial adherence to corneocytes compared to dogs and people, which could suggest relative resistance to the development of pyoderma in feline patients [33]. A study evaluating the frequency of isolation of Staphylococcus species bacteria in cats with and without inflammatory skin disease did not find a significant difference in bacterial isolation between the two populations [34]. However, other sources suggest that feline bacterial pyoderma may be more prevalent than previously reported [35,36]. As with NFNFHD, bacterial pyoderma in cats has less well-defined lesions than canine bacterial pyoderma. Variable lesions of feline bacterial pyoderma may include crusted papules with or without erosion and exudation, pustules, furuncles, eroded to ulcerated plaques which may also be crusted and exudative, and linear to nodular granuloma lesions that may be ulcerated [35]. As several of these lesions are similar to common cutaneous reaction patterns in feline NFNFHD, the involvement of bacterial infection may frequently be overlooked.

As with bacterial pyoderma, Malassezia species overgrowth is considered to be a common concurrent finding in dogs and people with AD. Historically, secondary Malassezia overgrowth in feline patients with allergic skin disease has only been reported anecdotally. More frequently, Malassezia overgrowth was noted in cats with systemic illnesses including thymoma-associated dermatitis, paraneoplastic alopecia [37], and cats infected with feline leukemia virus or feline immunodeficiency virus [38]. These reports led to the general belief that Malassezia overgrowth in the cat was an indicator of serious systemic disease including neoplasia or retroviral infections. As with bacterial pyoderma, however, secondary Malassezia overgrowth in cats with allergic skin disease may be a more prevalent finding than what was previously reported. One study found that Malassezia organisms were isolated much more frequently in cats with NFNFHD than in cats without allergic skin disease [39]. Lesions were in parallel with the distribution of feline allergic pruritus and were frequently accompanied by alopecia, erythema, greasy brown scales that adhered to the skin, increased cerumen, and hyperpigmentation. The improvement noted with administration of systemic antifungal medication (i.e., itraconazole) supported the contribution of Malassezia overgrowth to the degree of noted pruritus in allergic cats [39]. These studies support the association between secondary infections in cats with allergic skin disease and highlight the need for utilizing diagnostics and therapy to identify and resolve secondary bacterial pyoderma and yeast overgrowth in allergic feline patients.

**Figure 1:**
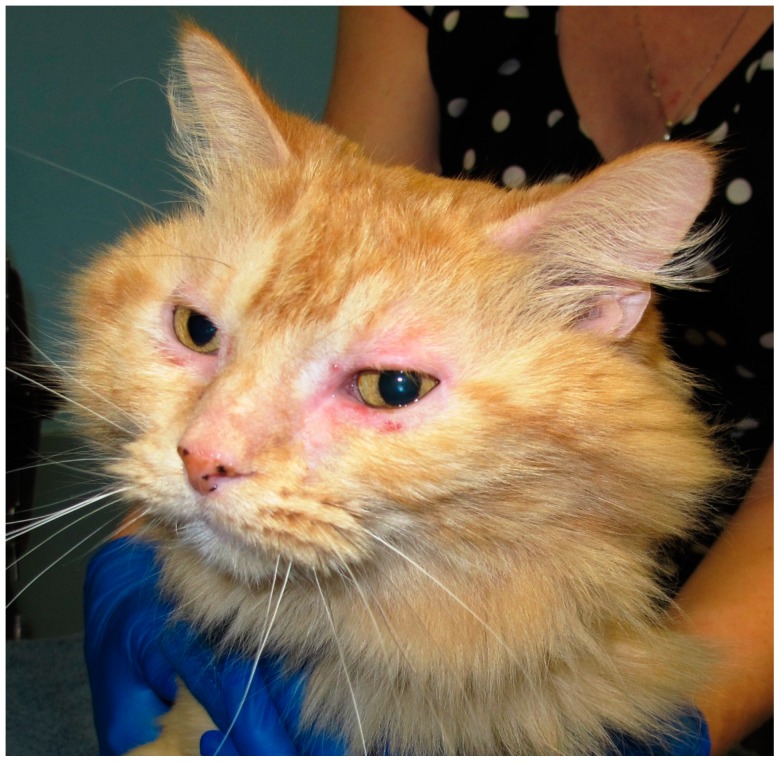
Cat with non-flea, non-food hypersensitivity dermatitis (NDNDHD). Allergic conjunctivitis, sinusitis, and facial pruritus were present.

Along with the cutaneous reactions patterns noted in cats with NFNFHD, other non-cutaneous clinical signs may be concurrently present. This may include allergic otitis, sinusitis, and conjunctivitis (Figure 1) as well as potentially feline small airway disease or “asthma”. However, the exact prevalence of these clinical presentations is not known.

Although pinnal pruritus is a fairly common manifestation of feline NFNFHD, the ear canals themselves are frequently normal in appearance. This is in contrast to atopic dogs who frequently present with erythematous otitis externa secondary to allergic disease [1]. Some cats with NFNFHD will present with recurrent, ceruminous otitis externa, often in the absence of infectious organisms (i.e., bacteria and yeast). This may be misdiagnosed as ear mite infestation even if mites are not found on mineral oil preparations. As cats tend to be a bit more sensitive to manipulation of the external ear canals, topical medicants and cleansers should be used with caution.

Sneezing may be a frequent concurrent clinical sign in cats with NFNFHD which may be indicative of sinusitis. Some sources cite prevalence in up to 50% of cats with NFNFHD [32]. As well, a single reported case of allergic rhinitis has been documented in the literature [40], but this may be more commonly seen clinically without a definitive (e.g., biopsy) diagnosis. The prevalence of concurrent sinusitis or rhinitis is not known since imaging studies have not been evaluated in cats with NFNFHD. Although considered to be a rare occurrence in allergic cats, conjunctivitis has also been reported [41]. However, the combination of these clinical signs may also be associated with other infectious causes such as feline herpes virus [42,43] or Mycoplasma felis infection [43]. It is important to rule out causes of feline upper respiratory tract infections even in the face of a clinical diagnosis of NFNFHD when sinusitis and conjunctivitis are present.

Feline small-airway disease or feline asthma is a complex syndrome; however, many cats have an allergic pathogenesis [44]. A pilot study demonstrated increased prevalence of positive reactions to aeroallergens in cats with small-airway disease compared to normal healthy cats on both intradermal and allergen-specific IgE serology tests [45]. Several additional studies have demonstrated the benefits of allergen-specific immunotherapy in cats with feline small-airway disease [46,47,48,49]. As with other non-cutaneous manifestations of feline allergy, the prevalence of concurrent small-airway disease in cats with NFNFHD is not known. In the pilot study evaluating the prevalence of positive reactions to inhaled allergens in cats with small-airway disease [45], the presence of concurrent or pre-existing dermatologic abnormalities was quite high, making recruitment of patients for the study difficult. This observation may indicate a higher percentage of cats with both allergic airway disease and NFNFHD; the severity of respiratory signs may overshadow the presence of concurrent skin disease or treatment for the small-airway disease (i.e., glucocorticoids) may control the signs of cutaneous allergy. Further study is warranted to better elucidate the relationship between the two disease conditions.

As with AD in other species, there is no cure for NFNFHD in cats. Rather, the goal of managing the disease relies on decreasing the severity and frequency of pruritic flares along with other cutaneous and non-cutaneous complications. Care should be taken to identify and manage any secondary disease entities such as parasites and infections as this will compound the degree of pruritus appreciated. Additionally, any other flare factors should be recognized and addressed.

Various therapeutic entities are available for managing feline NFNFHD. As no one specific treatment may be ideal, each option should be carefully evaluated for each feline patient. Medical therapeutic treatments to address the signs and symptoms associated with feline NFNFHD can be broken into conservative options and more aggressive medical therapy. Conservative treatment options include fatty acid supplementation and antihistamines [50]. More aggressive therapy includes administration of glucocorticoids or cyclosporine [50,51]. Cats tend to be a bit less sensitive to steroids compared to dogs and often require a higher starting treatment dose (e.g., 1–2 mg/kg prednisolone every 24 h and then tapered) [50]. The microemulsion form of cyclosporine (cyclosporine (modified); e.g., Atopica^®^, Elanco) is recommended in veterinary species due to more predictable absorption and bioavailability [52]. Oclacitinib (Apoquel^®^, Zoetis), a Janus kinase inhibitor labeled for the treatment of allergic pruritus and AD in dogs, has shown efficacy in reducing clinical signs in a small population of cats with NFNFHD [53]. This medication, however, has not been well studied yet in cats; ideal dose range and administration recommendations have yet to be established in this species. The caninized monoclonal antibody directed against interleukin-31 (lokivetmab; Cytopoint^®^, Zoetis) for the management of canine AD is not recommended for cats due to the likelihood of severe adverse reactions to the foreign protein [54]. Allergen-specific immunotherapy may also be an effective treatment options for cats with NFNFHD [55]. Various options for formulation exist; however, subcutaneous injection has been used most frequently in this species as compared to oral/sublingual allergy drops. Conventional induction as well as rush induction has been successful for cats with NFNFHD [56]. It is important to consider that allergen testing (e.g., intradermal or serum based) does not diagnose allergic skin disease in animal species; these tests should be used as a guide for immunotherapy formulation once a clinical diagnosis has been established.

## 5. Conclusions

Cutaneous hypersensitivity diseases in cats, while showing parallels to the similar allergic diseases in dogs, presents with several differences in regards to clinical signs, pathogenesis, and specific treatment recommendations. As with dogs, it is important to identify and address secondary complications of pruritus including ectoparasites and infections prior to embarking down the journey of working up the allergic feline patient. Imperative to keep in mind is that concurrent allergic diseases may present in the same patient. Addressing flea bite hypersensitivity and other insect allergies as well as food allergy prior to evaluating non-flea, non-food hypersensitivity dermatitis is key to successful disease control. Once a diagnosis of NFNFHD is reached, continuously assessing the presence of secondary complications and flare factors as well as evaluating options and differences in therapeutic recommendations will lead to improved patient care and disease management.

## Abbreviations

The following abbreviations are used in this manuscript:

IgE	immunoglobulin E
ENFNFHD	non-flea, non-food hypersensitivity
FAD	flea allergy dermatitis
FSA1	flea salivary antigen 1
DNA	deoxyribonucleic acid
DEET	*N,N*-diethyl-*m*-toluamide
CAFR	cutaneous adverse food reaction
AD	atopic dermatitis
CD	cluster of differentiation molecule
